# Supplementary material for: On the emergence of P-Loop NTPase and Rossmann enzymes from a Beta-Alpha-Beta ancestral fragment
Source: eLife. 2020 Dec 9;9:e64415. doi: 10.7554/eLife.64415 (PMC7758060; doi:10.7554/eLife.64415)
Supplement: Supplementary file 1. [file elife-64415-supp1.docx]

**Supplementary File 1: Dication binding in tubulins**

| **PDB ID** | **Chain ID** | **Tubulin Subfamily** | **Bound Metal** | **β2 Residue** | **Distance to Bound Metal^*^** |
| --- | --- | --- | --- | --- | --- |
| 4F6R | A | Alpha-beta tubulin | Mg | Asp | 3.9 |
| 4FFB | A | Alpha-beta tubulin | Mg | Asp | 4.2 |
| 4I4T | A | Alpha-beta tubulin | Mg | Asp | 4 |
| 4U3J | A | Alpha-beta tubulin | Mg | Asp | 4 |
| 5IYZ | A | Alpha-beta tubulin | Mg | Asp | 4.1 |
| 5NQU | A | Alpha-beta tubulin | Mg | Asp | 4 |
| 6GWC | A | Alpha-beta tubulin | Mg | Asp | 4.1 |
| 1W58 | A | FtsZ | Mg | Asn | 4.7 |
| 1W5A | A | FtsZ | Mg | Asn | 4.5 |
| 1W5F | A | FtsZ | Mg | Asn | 4.5 |
| 2R75 | A | FtsZ | Mg | Asn | 4.3 |
| 1Z5V | A | Gamma tubulin | Mg | Asp | 3.7 |
| 1Z5W | A | Gamma tubulin | Mg | Asp | 4 |
| 3RB8 | A | PhuZ | Mg | Asp | 3.9 |
| 2XKA | A | TubZ | Mg | Asn | 5.3 |
| 2XKB | A | TubZ | Mg | Asn | 5 |

^*^Taken as the distance between the metal and the closest sidechain oxygen of the β2-Asp or the sidechain oxygen for the equivalent Asn.
